# Dynamic Behavior of Advanced Materials and Structures

**DOI:** 10.3390/ma18122878

**Published:** 2025-06-18

**Authors:** Weidong Song, Lijun Xiao

**Affiliations:** State Key Laboratory of Explosion Science and Safety Protection, Beijing 100081, China; xljbit@bit.edu.cn

Following the rise in applications of materials and structures in complex environments, such as high-speed impacts and explosions, research on the dynamic response of materials and structures is becoming increasingly important [1,2,3]. Studying their mechanical behavior of advanced materials and structures, such as mechanical metamaterials [4,5,6,7,8], composites [9,10,11], and lightweight sandwich structures [12,13,14], under dynamic loads and gaining a thorough understanding of their behavioral characteristics under extreme conditions are vital for both material and structural optimization, as well as ensuring their safety and reliability in service. This Special Issue (SI), “Dynamic Behavior of Advanced Materials and Structures”, presents recent theoretical, experimental, and simulation research findings regarding the dynamic behavior of advanced material and structures. This Editorial summarizes the ten publications (nine research articles and one review article) included in this SI.

Unlike traditional materials that expand laterally under compression, auxetics display a distinct negative Poisson’s ratio characteristic, which enhances their energy absorption capacity under dynamic loading [15,16,17]. Accordingly, auxetics have attracted extensive research attention in recent years [18,19,20]. Galea Mifsud et al. [21] summarized the research progress on analyzing the mechanical properties and deformation mechanisms of auxetic systems through finite-element simulations.

Carbon-fiber-reinforced polymers (CFRPs) have a variety of advantages properties in comparison to metals, including light weight, high strength, high stiffness, corrosion resistance, and fatigue resistance [22,23], and have been widely used in fields of aerospace and traffic control. However, the dynamic performance and failure mechanism of CFPRs remain unclear due to their complex mesoscopic structure. Qiang et al. [24] presented the low-velocity impact response and residual compressive properties of carbon fiber–epoxy resin (CF/EP) composites, revealing the impact damage on the compression failure mechanisms of the materials. Xi et al. [25] reported that the energy absorption capacities and failure modes of the open-section, thin-walled composite structures under axial crushing loading could be regulated by changing the geometry of the cross-section. Stachyra et al. [26] introduced a novel analytical description for coupled mode shapes of a cantilever composite beam. Lan et al. [27] demonstrated that the preloading value and the impact velocity affected the delamination behavior of CFRP laminated plates significantly. The resistance to out-of-plane displacement of the laminated plates could be enhanced by biaxial tensile preload.

With the advancement of technology, lightweight materials have become widespread in the development of protective structures. Sandwich structures filled with lightweight materials, such as foams and honeycombs, have been widely applied in energy-absorbing devices for collision protection in fields including aerospace, rail transportation, and the automotive industry, due to their light weight, high specific strength, and high specific stiffness [28,29]. How to design high-efficiency sandwich structures for energy absorption is a widely studied topic. Mahgoub et al. [30] experimentally investigated the dynamic response of a sandwich beam filled with a stepwise gradient polymethacrylimide (PMI) foam core under low-velocity impacts, demonstrating that a negative gradient core is beneficial for energy absorption.

In military and public security safety fields, materials and structures are often subjected to explosive shock waves. Bian et al. [31] investigated the dynamic response of non-metallic annular protective structures made by the continuous winding of PE fibers under internal blasts, indicating that the blast resistance of the structure can be improved by adding polyurethane foam in the inner layer. The dynamic response of a rectangular steel plate subjected to shock waves was examined by Li et al. [32] through the wavelet transform (WT) and the improved ensemble empirical mode decomposition (EEMD) methods.

Steel automotive wheel rims typically experience dynamic wear and tear during service, which significantly affect their service life. Borecki et al. [33] proposed new methods for identifying the technical condition of steel car wheel rims and predicting the approaching end of wheel rim service life with limited parameters.

Electromagnetic springs, which act as an active vibration isolation system, have been widely researched due to their fast response, non-contact, and adjustable stiffness [34,35]. In the study by Zheng et al. [36], an improved Bouc–Wen Model was proposed to describe the dynamic characteristics of a toothed electromagnetic spring, which enhanced the accuracy of predicting the hysteresis behavior in electromagnetic spring active isolators.

Nowadays, with the rapid development of advanced manufacturing technologies such as additive manufacturing, an increasing number of advanced materials and structures have been developed, including multi-component alloys [37,38,39], interpenetrating phase composites [40,41,42], and nanomaterials [43,44]. Future research needs to pay more attention to the dynamic behavior of these novel materials. Simultaneously, the dynamic behavior of materials often depends on their microstructures, necessitating more extensive application of multi-scale analysis methods for investigation [45,46,47]. Additionally, integrating artificial intelligence technologies with mechanical design and the prediction of materials/structures [48,49,50] also requires further investigation.
